# Immunotherapy of Equine Sarcoids—From Early Approaches to Innovative Vaccines

**DOI:** 10.3390/vaccines11040769

**Published:** 2023-03-30

**Authors:** Christoph Jindra, Edmund K. Hainisch, Sabine Brandt

**Affiliations:** 1 Division of Molecular Oncology and Hematology, Karl Landsteiner University of Health Sciences, 3500 Krems an der Donau, Austria; 2 Research Group Oncology (RGO), Division of Equine Surgery, Department for Companion Animals and Horses, University of Veterinary Medicine, 1210 Vienna, Austria

**Keywords:** horse, equids, sarcoid, bovine papillomavirus, BPV1, BPV2, immunotherapy

## Abstract

Horses and other equid species are frequently affected by bovine papillomavirus type 1 and/or 2 (BPV1, BPV2)-induced skin tumors termed sarcoids. Although sarcoids do not metastasize, they constitute a serious health problem due to their BPV1/2-mediated resistance to treatment and propensity to recrudesce in a more severe, multiple form following accidental or iatrogenic trauma. This review provides an overview on BPV1/2 infection and associated immune escape in the equid host and presents early and recent immunotherapeutic approaches in sarcoid management.

## 1. Introduction

Horses and other equid species such as donkeys, mules or zebras are frequently affected by skin tumors termed sarcoids. These tumors do not metastasize. Nonetheless, they constitute a serious health issue due to their resistance to therapy and their propensity to progress from mild-type occult or verrucous to more aggressive nodular, fibroblastic, mixed, or malevolent lesions that may affect a considerable proportion of the integument [1,2]. Sarcoids are also an economically relevant disease. They may impair the use and resale value of affected animals due to their location and clinical presentation, e.g., as multiple, bleeding masses [2]. As a result, these non-malignant skin tumors constitute the major dermatological reason for euthanasia in equine medicine [3].

## 2. Association of Bovine Papillomaviruses with Sarcoid Disease

Papillomaviruses (PVs) are a family of small non-enveloped viruses that can induce cutaneous or mucosal lesions ranging from benign warts to cancer in humans and animals. Despite their pronounced genetic diversity, all PVs have similar structures. All PV virions consist of an icosahedral capsid harboring a circular double-stranded DNA genome of <8 kbp in length [4]. This genome codes for early (E) regulatory (e.g., E1, E2) and transforming proteins (e.g., E6, E7, E5) and the late (L) capsid proteins L1 and L2 [5]. In addition, all PV genomes contain a non-coding long control region (LCR) downstream of the L1 open reading frame (ORF). This region contains cis-responsive elements that are necessary for viral replication and transcription [5]. Early research in animal PV models has revealed that most PVs are epitheliotropic and highly species-specific. The genus of delta- (δ-) PVs does not adhere to this general rule. Its members also infect dermal fibroblasts, and probably for this reason have a wider host range [4,6]. This is best documented for bovine δ-PV types 1 and 2 (BPV1) that infect not only cattle but also other ungulates, including equid species [2]. 

**Figure 1:**
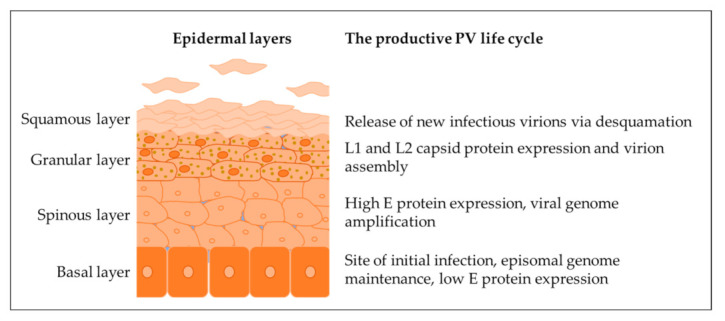
The productive PV life cycle is tightly linked to the differentiation program of infected keratinocytes.

The productive life cycle of PVs is restricted to epithelial keratinocytes, and its organization is tightly linked to the stages of keratinocyte differentiation and migration to the epithelial surface, as schematically represented in Figure 1. Throughout the productive life cycle, PV infection is episomal with multiple copies of genomic DNA residing in cell nuclei in an extrachromosomal form. However, PV infection is not always productive. In cases of PV-mediated transformation, keratinocytes can remain in a poorly differentiated state, which does not support virion production. Consequently, infection is non-productive, with viral episomes efficiently replicating in synchrony with cell division [5]. Viral DNA can also integrate into the host cell genome as shown for carcinogenic human PVs (HPV) termed high-risk HPVs (hrHPVs) [5]. This event is commonly associated with tumor progression [7,8,9]. 

BPV1 and BPV2 are closely related δ-PV types as demonstrated by their >90% identity on protein level. In cattle, infection by these virus types commonly leads to the development of benign fibropapillomas (warts) that regress spontaneously in immunocompetent individuals. In the epidermal portion of the lesions, infection is productive, with high numbers of new virions being produced and released via desquamation. In the dermal portion of the lesions, infection of fibroblasts is exclusively episomal [6]. In horses and other equids, first evidence of a causal association of BPV1/2 infection with sarcoid development was provided in the 1950s, when Olson and Cook succeeded in inducing sarcoid-like lesions via inoculation of skin with cell-free bovine papilloma extract. These lesions termed pseudo-sarcoids were morphologically and histopathologically indistinguishable from natural sarcoids, but resolved over time [10]. The advent of sophisticated molecular biological and immunological techniques allowed sarcoid research on molecular level and paved the way for the recognition of BPV1 and BPV2 as the major causative agents of sarcoids in horses and other equids [11]. In addition, there are indications of closely related bovine δ-PV type 13 causing sarcoids [12,13]. 

In equids, many aspects of BPV1/2 infection are still unclear. This applies, e.g., to virus transmission, which is thought to occur by direct contact with infected animals and contaminated material, and possibly flying insects [11]. Moreover, BPV1/2 infection was for a long time thought to be exclusively episomal and restricted to dermal fibroblasts [2,11,14]. However, more recent data indicate that BPV1/2 infection in horses can also involve the epidermis, with low amounts of virions being (occasionally) produced [15,16,17]. Interestingly, BPV1 and BPV2 can establish latent episomal infection also in apparently healthy skin of sarcoid-affected horses [16,18,19,20,21]. This likely explains the frequently reported onset, progression, or reoccurrence of sarcoids following accidental or iatrogenic trauma [11,22,23,24]. The role of trauma in (re-) activation of PV-induced disease is widely accepted today [25]. However, it remains completely unclear how BPV1/2 infection can spread within the equid’s body and eventually involve the entire integument. 

BPV1/2 infection of equid fibroblasts cause pathobiological changes that mainly include sustained hyperproliferation, loss of contact inhibition, and resistance to apoptosis [26,27,28]. Ex vivo investigations and in vitro studies in primary sarcoid cells, as well as BPV-transfected and -infected primary equine fibroblasts have considerably helped understand BPV1/2 oncoprotein-mediated cell transformation. In transfected equine fibroblasts, it was shown that BPV1 E5 and E6 synergistically induce morphological changes (i.e., an elongated spindle-shaped phenotype), promote hyperproliferation, and contribute to invasive growth. In addition, E6 and E7 were shown to confer anchorage independence to growing fibroblasts [27,28]. In agreement with these findings, experimental infection of equine fibroblasts with wild-type BPV1 or BPV2 virions resulted in sustained hyperproliferation and loss of contact inhibition as evidenced by cells piling up and forming spheres. From passage 4 until final passage 34, infected cells harbored constant levels of viral episomes and gene transcripts, suggestive of infection-mediated immortalization [26]. Cellular immortality is essentially mediated by telomerase activity. Interestingly, BPV2-induced bovine cancers, BPV1-associated equine sarcoid cells, and BPV1 E6-transfected equine fibroblasts were reported to equally express telomerase [27,29]. The consensus of these findings and the evidence that telomerase is activated by HPV type 16 E6 [30] suggest that in vitro and in vivo immortalization of infected equid fibroblasts is chiefly mediated by BPV1/2 E6. Functional in-depth analyses on molecular level uncovered major mechanisms underlying abovementioned growth characteristics of BPV1-infected cells. These include the ability of E5 to activate the platelet-derived growth factor (PDGF) β-receptor [31,32] of E6 to interact with the focal adhesion protein paxillin [33], and of E7 to bind to p600 [34].

## 3. BPV Immune Escape in the Equid Host

In the bovine host, BPV1/2 infection is associated with benign fibropapillomas that usually regress after several months. Whilst the humoral response to BPV1/2 infection is limited, a cellular immune response ultimately leads to fibropapilloma rejection [6]. In equids, the immune response to BPV1/2 infection and associated sarcoids is still poorly understood [35]. Intramuscular administration of BPV1 L1 virus-like particles was shown to induce high-titer neutralizing antibodies conferring efficient protection from experimental infection with wild-type BPV1 virions [36,37]. In contrast, neither natural infection in sarcoid-bearing horses nor experimental infection of healthy horses with wild-type BPV1 or BPV2 virions induce a significant antibody response [38,39]. Yet, like bovine fibropapillomas, experimental pseudo-sarcoids spontaneously regress [10,38,40,41], indicating that rejection of BPV1/2-associated lesions is largely mediated by a cellular immune response induced by unnaturally high virion concentrations. This response is likely impaired in naturally sarcoid-affected equids, which may explain the usual persistence of disease [2].

BPV1/2 infection in equids is restricted to the skin and mainly involves dermal fibroblasts where virus resides in an episomal form. Infection is associated with neither viremia nor cytolysis, cell death or inflammation, thus significantly impeding immune recognition [2,42]. Several other immune evasion mechanisms likely contribute to persistence of BPV1/2 infection and ensuing sarcoids.

The Innate immune response constitutes the first line of defense against invading pathogens. Toll-like receptors (TLRs) are crucial to this response as they recognize conserved pathogen motifs termed pathogen-associated molecular patterns (PAMPs). Importantly, Yuan and colleagues have shown that BPV1 E2 and E7 proteins downregulate TLR4 transcription in transformed equine fibroblasts and sarcoid cells, thus impairing the production of cytokines and adhesion molecules required for BPV1 control [43,44]. 

E5 is the major oncoprotein of BPV1 and BPV2, and is consistently expressed in sarcoid cells [2,45]. In addition to its transforming activity, E5 chiefly contributes to viral immune evasion by downregulating the major histocompatibility complex class I (MHC I) on the transcriptional level and during its trafficking to the cell surface [44,46]. Given that antigen recognition by CD8^+^ T lymphocytes is MHC-restricted, E5-mediated MHC I suppression undermines adaptive defense mechanisms that rely on antigen recognition and clearance by cytotoxic T cells (CTLs) [46,47]. In addition, bovine δ-PV E5 proteins were recently shown to impair innate immune signaling pathways mediated by RIG-I-like receptors and cGAS-STING [48,49].

Genetic studies conducted in different horse families and breeds revealed a significant association of specific equine MHC I and MHC II variants with the risk to develop sarcoids upon BPV1/2 infection [2,50,51,52]. This notably applies to the MHC I haplotypes A5 and B1, and the MHC II haplotypes W3 and W13 [50,51,52]. Given the importance of MHC-mediated antigen presentation to T cells, genetically divergent MHC I and MHC II alleles may alter the functionality of these molecules and hamper BPV antigen processing and recognition. 

Foxp3 belongs to the family of forkhead/winged-helix transcription factors that regulate the development and function of the immune system. Foxp3 is crucially involved in the generation of CD4^+^CD25^+^ regulatory T cells (Tregs). Accordingly, loss of function of this molecule entails lack of Tregs ensuing lethal autoimmune disease. In contrast, Foxp3 overexpression induces Treg-mediated immunodeficiency [53]. Under physiological conditions, Tregs ensure the maintenance of immunological self-tolerance and homeostasis. In different types of cancer, however, Tregs create an immunosuppressive tumor environment promoting disease progression [54,55]. Interestingly, equine sarcoids were shown to harbor high numbers of CD4/CD8 double-positive cells uniformly co-expressing Foxp3. These cells assumingly represent T lymphocytes with a regulatory function [56]. However, Geisshüsler et al. detected CD4^+^ Foxp3^+^ RORγt^-^ Tregs in sarcoids and normal skin at similar proportions, whilst pro-inflammatory CD4^+^ Foxp3^+^ RORγt^+^ Tregs were downregulated in sarcoid tissue [57]. Interestingly, sarcoids likely exhibit a regulatory cytokine environment, as revealed by upregulated transcription of transforming growth factor β (TGFβ) and interleukin 17 (IL17). This environment is thought to prevent inflammation in sarcoid tissue [56].

Intriguingly, there is increasing evidence that Foxp3 is expressed not only by blood cells, but also by different types of tumor cells [53]. For example, Foxp3 has been detected in pancreatic ductal adenocarcinoma (PDAC), melanoma, hrHPV-induced cervical cancer, and—importantly—also sarcoid cells [56,58,59,60]. Expression of Foxp3 in human cancer cells and BPV1-infected sarcoid fibroblasts raised the question of whether Foxp3 may confer immune regulatory functions to these cells. First evidence for such a scenario was provided in 2017, when Wang and colleagues reported on the efficient recruitment of Tregs by Foxp3^+^ PDAC cells via transactivation of the chemokine CCL5 [60]. Investigations are warranted to elucidate whether Foxp3^+^ sarcoid cells recruit Tregs in a similar manner, thus contributing to the creation of protumoral immune milieu in the lesions.

## 4. Immunotherapy of Sarcoids

Given the high veterinary and economical significance of sarcoid disease in equid populations, efforts have been made for many years to develop more effective therapeutics. These include immunotherapeutic approaches, which aim at stimulating the immune system to regain control of PV infection and associated tumor development. Immunotherapy of sarcoids is still in its infancy and only a few approaches have been shown to be beneficial so far. These include Toll-like receptor agonists, immunostimulatory cytokines, recombinant virus-like particles, and autologous implantation [23,61,62,63]. In addition, interesting data on a new virus vector-based vaccine have been provided recently [64]. 

### 4.1. Toll-like Receptor Agonists 

**Figure 2:**
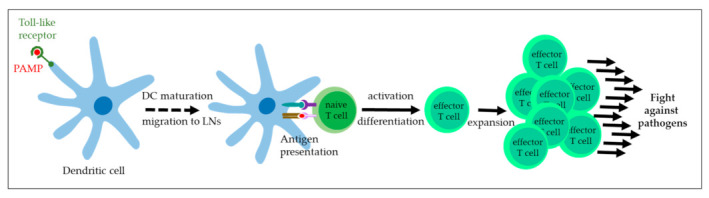
Role of Toll-like receptors in T cell activation. Recognition of PAMPs by TLRs expressed by dendritic cells (DCs) leads to maturation of these antigen-presenting cells and their migration to draining lymph nodes. In the presence of co-stimulatory molecules, DCs present antigens via MHC molecules to naive T cells. Upon this stimulus, naive T cells differentiate into effector T cells that expand, move to the site of infection, and fight the invading pathogens [65,66].

The first line of defense against incoming pathogens involves the innate immune system. Upon interaction with pattern-recognition receptors (PRRs), immune cells such as monocytes and macrophages, dendritic cells (DCs), neutrophils, and natural killer cells (NKs) recognize PAMPs or damage-associated molecular patterns (DAMPs) and secrete type I interferons (IFNs) and co-inflammatory cytokines as mediators of defence. TLRs also have an important role in T cell activation, as illustrated in Figure 2 [65,66]. The family of PRRs comprises TLRs that are expressed by immune but also epithelial cells to confer protection from pathogens to cutaneous and mucosal surfaces [67].

Bacillus Calmette-Guérin (BCG), a live-attenuated Mycobacterium bovis derivative, was initially developed for prevention of tuberculosis [68]. The discovery of its antitumor activity via TLR2- and TLR4-medidated immune stimulation paved the way for the use of BCG in cancer therapy [69]. First reports on BCG-based sarcoid treatment date from the 1970s, describing tumor regression in several cases following intralesional BCG injection [70,71]. BCG is mainly used in the form of cell wall extract in oil, and is injected one to several times into the tumor. Overall, there is agreement that BCG can be effective in the treatment of sarcoids [23,72,73,74]. Good results are notably reported for periocular lesions for which the therapeutic repertoire is generally limited [23,73,75,76,77]. Knottenbelt and Kelly reported on a 69% regression rate for nodular and fibroblastic lesions following intratumoral BCG administration, whilst occult and verrucous sarcoids only poorly responded to this type of treatment [73]. This partly contrasts with the reported observation that BCG was most effective in the therapy of small, solitary lesions [77]. Interestingly, BCG-based treatment of sarcoids affecting the distal limbs seems to be less beneficial and more frequently associated with complications, so that use of BCG in such cases is not recommended [78]. 

When BCG treatment was compared to other therapeutic modalities such as surgical excision (conventional or laser), radio-, cryo-, or chemotherapy, the performance of BCG was similar or inferior to these other approaches [73,74]. This evidence and the difficulty/impossibility to purchase BCG in many countries [79] likely explain the infrequent use of BCG in today’s equine practice.

The signaling cascade induced by TLR-mediated recognition of invading pathogens is evolutionarily conserved among mammals. In mice, Hemmi et al. demonstrated that the antiviral and antitumor effect of the imidazoquinoline compound imiquimod is based on the activation of immune cells via TLR7. The success of imiquimod in the treatment of HPV-induced non-cancerous lesions as reviewed by Kollipara and colleagues [80] has also led to its use in sarcoid therapy. In a pilot study involving fifteen horses, repeated topical application of imiquimod 5% cream on sarcoids resulted in complete tumor regression in 60% of cases within a period of 32 weeks. Interestingly, a tendency towards nodular lesions showing the best and fibroblastic tumors exhibiting the poorest response to treatment was noted. Adverse side effects such as exudation, erythema, erosions and alopecia were limited to the application site [81]. In a similar study, treatment of sarcoids with imiquimod 5% cream was effective in 84.4% of sarcoid cases, with fibroblastic sarcoids showing the highest level of therapy resistance in agreement with previous observations [82]. The safety and efficacy of imiquimod 5% cream in sarcoid treatment is also described by Haspeslagh et al. [83]. Use of this TLR7 agonist thus constitutes a promising therapeutic approach, especially in cases where other therapeutic interventions are not indicated or not affordable by owners.

### 4.2. Immunostimulatory Cytokines

Upon recognition of PAMPs or DAMPs, TLRs initiate a signaling cascade that comprises the synthesis and secretion of cytokines as key mediators of immune cell activity. In hrHPV-induced cervical cancer and HNSCC, expression of immunostimulatory T helper 1 (Th1) cytokines such as interleukin 2 (IL-2), IL-12, tumor necrosis factor α (TNF-α), and IFNs is downregulated, whilst Th2 proinflammatory and immunosuppressive cytokines are overexpressed [84,85,86,87,88]. This imbalance leads to the development of immunotherapeutic approaches aiming at reestablishing an antitumoral and antiviral Th1 cytokine profile [84]. In human medicine, various cytokine-based treatments such as injectable immunostimulatory cytokines are currently being evaluated for several tumor diseases including hrHPV-induced cancers [84,89,90]. In equine medicine, only data on the use of IL-2-based therapeutics are available so far. 

IL-2 is predominantly expressed by antigen-activated CD4^+^ T cells, but also CD8^+^ T lymphocytes and other immune cells such as NK T cells and monocytes. The antitumor effect exerted by IL-2 is initiated by its binding to the multimermeric IL2 receptor (IL2-R), which elicits a signal transduction cascade, ultimately leading to the maturation and activation of DCs, stimulation of NK cell cytotoxicity, expansion of CD4^+^ and CD8^+^ T lymphocytes, and Th1 polarization of the immune response [84,90,91]. On the other hand, IL-2 also has an essential role in prevention of autoimmunity by promoting Treg maturation and expansion. This feature and the dose-limiting toxicity of systemically administered Il-2 explain its limited use in human cancer therapy [91]. 

In veterinary medicine, local administration of low-dose IL-2 is described as being safe and effective in the therapy of several experimental and natural tumors, including bovine ocular SCC [92]. Spoormakers and colleagues were likely the first to use IL-2 in sarcoid therapy. Repeated intratumoral injections of low-dose IL-2 or single high-dose IL-2 combined with cisplatin resulted in complete tumor regression in 14% and 53% of cases. This finding pointed to low-dose IL-2 being rather ineffective in the treatment of sarcoids [93]. In another clinical trial, 20 horses with a total of 59 sarcoids were treated by two intratumoral injections (day 0, day 7) with recombinant canarypox virus co-expressing feline IL-2 (fIL-2). Whilst authors provided no evidence of IL-2 being expressed in the injected lesions, treatment resulted in complete tumor regression in eight, and partial regression in two horses [94]. Four intratumoral doses (day 0, after 1, 3, and 7 weeks) of the therapeutic vaccine led to complete sarcoid regression in 7/14, and partial regression in 5/14 horses [95]. Of note, response to treatment inversely correlated with severity of disease in both studies. Given its good safety profile, the canarypox-based fIL-2 vaccine may be applied in the treatment of single mild- to moderate-type sarcoids [94,95]. 

### 4.3. Recombinant Virus-like Particles

The discovery that in vitro-generated PV capsid proteins spontaneously self-assemble to highly immunogenic capsids termed virus-like particles (VLPs) has led to establishment of vaccines for protection against infection by high- and low-risk HPV types [96,97]. Similarly, BPV1 L1 VLPs have proven effective in protecting horses from experimental BPV1 and BPV2 infection [36]. On the other hand, PV L1 (and L2) VLPs have no therapeutic effect, as exemplarily shown for BPV4 VLPs in calves bearing BPV4-induced palatal papillomas [98]. To overcome this limitation, Ashrafi et al. generated chimeric BPV1 L1 VLPs also containing E7 peptides (CVLPs) as antigens. In an efficacy study, sarcoid-bearing donkeys received the vaccine or placebo (PBS) at days 0, 14, 35, 49, 70, 95 and 112 through intramuscular injection. Although a tendency towards regression and reduced progression of some of the CVLP-treated lesions was noted, no significant therapeutic effect could be reached [61]. Similar results were obtained in sarcoid-affected horses, where sarcoid progression rather than regression was observed in twelve animals following CVLP treatment. Moreover, only 5/12 sarcoid-bearing horses seroconverted to the E7 component of the vaccine [63].

### 4.4. Autologous Vaccination

Autologous vaccines are produced from excised sarcoid material that is cut into small pieces, wrapped in gauze, repeatedly frozen by complete immersion in liquid nitrogen, and then reimplanted subcutaneously into the sarcoid patient [99]. The idea underlying this approach is to re-instruct the patient’s immune system to recognize and fight BPV1/2 infection and resulting tumor disease [99,100]. In a study involving 15 sarcoid-affected horses, 12/15 sarcoids regressed within 90 to 180 days following reimplantation, whilst three horses failed to respond [99]. In another study, treatment of 16 horses with single or multiple sarcoids by autologous vaccination resulted in owners reporting on a decrease in sarcoid numbers in 75% and of sarcoid sizes in 93.8% of cases. Complications were noted in 7/16 horses and mainly consisted of swelling at the implantation site, followed by fever and abscess formation [100]. These reported findings are in agreement with the authors’ clinical experience: In one referred case, growth of sarcoids at 2/4 implantation sites was noted, and likely caused by inefficient freezing of the implants by the referring veterinarian using cryotherapy spray (EKH, personal observation). The same phenomenon was also observed in 2/20 cases where implant inactivation was carried out by immersion in liquid nitrogen [101].

### 4.5. Influenza Virus Vector-Mediated Immunotherapy

Influenza (Flu) viruses belong to the Orthomyxoviridae family. They are enveloped icosahedral viruses of 80 to 120 nm in diameter harboring a segmented negative-sense RNA genome. The eight RNA segments of Flu viruses A and B code for ten proteins including the non-structural protein NS1. The latter acts as IFN antagonist, thus allowing Flu viruses to abrogate immune defense and establish infection in host cells [102]. In concordance, NS1-deleted Flu A and B viruses are live-attenuated and can only replicate in IFN-deficient systems such as Vero cells [102,103]. Importantly, NS1-deleted Flu viruses are highly immunogenic, as reflected by induction of high-titre neutralizing antibodies [104,105] and activation of CTLs and NK cells [106,107]. In addition, the truncated NS1 ORF allows for insertion of biologically active foreign genes [108,109,110,111,112]. This possibility was recently exploited for the generation of immunotherapeutic vaccines targeting PV-induced malignancies. In mice, injection of established TC1 tumors with NS1 deleted Influenza A viruses co-expressing inactivated HPV16 E6 and E7 peptides led to complete regression of 50% and partial regression of 25% of lesions [113]. Based on these findings, partially NS1-deleted Flu A and B viruses co-expressing shuffled BPV1 E6 and E7 peptides were generated and their safety confirmed in the horse. Subsequently, the therapeutic efficacy of the vaccine was addressed in 29 horses with mild (n = 5), moderate (n = 5) or severe sarcoid disease (n = 19) [64]. Treatment was carried out by repeated injections of a single lesion or selected tumors in case of multiple lesions with the Flu A and/or the Flu B-based vaccine, as outlined in Table 1.

**Figure 3:**
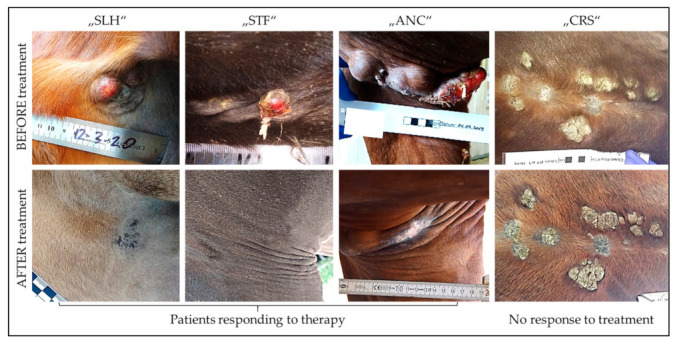
Influence of clinical sarcoid types on response to immunotherapy.

Immunotherapy led to complete regression of injected and non-injected tumors in 100% of mildly, 40% of moderately, and 31.5% of severely sarcoid-affected horses (Table 1). Complete disease resolution is expected in several cases where regression is still ongoing (=PAR). Two of the three moderately affected horses with still regressing sarcoids (Table 1; PAR) have been sold, so that no follow-up is available. There were no indications of differences in performance regarding the treatment modalities employed (Table 1). However, the low number of patients per treatment group did not allow conclusions with respect to the optimum therapeutic scheme. Unsurprisingly, horses with mild disease best responded to therapy. However, severity of sarcoid disease was not identified as major limiting factor, since complete tumor regression was also achieved in one third of patients with multiple sarcoids of various type classified as severe disease (Table 1) [64]. It rather appeared that clinical sarcoid presentation considerably influenced the therapeutic outcome: whilst most horses with single or multiple occult, nodular, and fibroblastic sarcoids responded well to treatment, all patients with multiple, extensive verrucous sarcoids resisted to therapy as exemplarily shown in Figure 3. The reasons for this phenomenon are unclear.

Treatment induced a systemic immune response as reflected by regression of injected as well as non-injected lesions in responding sarcoid patients [64]. Importantly, scrapings collected from previous tumor sites following complete disease remission scored negative by BPV1/2 PCR in 9 of 10 horses so far. This finding underscores the BPV1/2 specificity of the immunotherapeutic vaccines and the potential of the latter to eradicate BPV1/2-infection underlying sarcoid development and recurrence following ineffective treatment (manuscript in preparation). PCR-based monitoring of responders for BPV1/2 infection is still ongoing.

## 5. Future and New Directions

Currently available therapeutic approaches focus on surgery and other local treatment strategies. Importantly, none of these methods target the underlying viral cause of sarcoid development. As a result, sarcoids often recrudesce in a more severe, multiple form following therapy. The immune evasive microenvironment of sarcoids can be mitigated using non-specific immune modulators such as imiquimod, BCG or IL-2. Yet, this effect is only local and of limited duration. Autologous vaccination and recombinant VLPs offer more specificity yet are often unable to induce an effective systemic immune response.

In contrast, live-attenuated Influenza viruses co-expressing inactivated BPV1 E6 and E7 are able to potently reinstruct the immune system and guide it towards the root of sarcoid disease, i.e., BPV1 and/or BPV2 infected cells. Vaccine-induced cellular immune response likely results in systemic eradication of BPV1/2-positive cells, thus offering an unprecedented opportunity for disease cure without apparent risk of recurrence. In severely sarcoid-affected horses, the vaccine may be combined with surgery or other tumor eradication techniques to clear remaining infected cells and thus reduce the risk of disease recurrence. In exclusive and combined application of the vaccine, the assumed induction of immunological memory should render successfully treated horses immune to future BPV infection. Research addressing this issue is in progress.

## Figures and Tables

**Table 1:** Treatment modalities and outcome as of March 2023.

Treatment Schedule	Used Vaccines	Number and Types of Cases	Therapeutic Outcome			
			COR	PAR	STD	PRD
Days 1, 3, 5/8, 10, 12	AAA/BBB	7 severe	2/7	-	2/7	3/7
		1 moderate	1/1	-	-	-
Every second month on average until COR or no further improvement	AAA	4 severe	1/4	1/4	-	2/4
		4 moderate	2/2	2/2 ^§^	-	-
		2 mild	2/2	-	-	-
	AA, then B *	3 severe	1/3	1/3	-	1/3
		1 mild	1/1	-	-	-
	BBB	3 severe	1/3	-	2/3	-
	BB, then A *	2 severe	1/2	-	1/2	-
		2 mild	2/2	-	-	-

A: Flu A-based BPV1E6E7 vaccine; B: Flu B-based BPV1E6E7 vaccine. COR: complete regression; PAR: partial regression; STD: stable disease; PRD: Progressive disease. * repeatedly applied; **^§^** monitoring had to be discontinued because horses were sold.

## Data Availability

Not applicable.
